# The Development of the Study of Hereditary Cancer in South America

**DOI:** 10.1590/1678-4685-GMB-2014-0366

**Published:** 2016-05-24

**Authors:** Benedito Mauro Rossi, Carlos Sarroca, Carlos Vaccaro, Francisco Lopez, Patricia Ashton-Prolla, Fabio de Oliveira Ferreira, Erika Maria Monteiro Santos

**Affiliations:** 1 Grupo de Estudios de Tumores Hereditarios, São Paulo, SP, Brazil; 2 Hospital Sírio-Libanês, São Paulo, SP, Brazil; 3 Hospital Militar, Montevideo, Uruguay; 4 Hospital Italiano, Buenos Aires, Argentina; 5 Clínica Las Condes, Santiago, Chile; 6 Hospital de Clínicas and UFRGS, Porto Alegre, RS, Brazil; 7 A.C. Camargo Cancer Center, São Paulo, SP, Brazil

In 1989, at the beginning of the information age and its possibilities for the organization of databases, we implemented a system to collect clinical, individual and family data when there was suspicion of hereditary predisposition to cancer. It was a success because before it had never been so easy to retrieve data files. This database was extremely helpful and boosted the registry and the study of these individuals and families. With the possibility of retrieving data, we could precisely know the number of cases of cancer in young patients and relate these with the history of cancer in the family.

As a consequence of the organization of information on patients and their families, we organized in 1992 the first Brazilian Hereditary Colorectal Tumor Registry. One of the first fruits of the Registry occurred in 1994 with the first molecular diagnosis of Familial Adenomatous Polyposis (FAP) in a Brazilian family with mutation in the *APC* gene, located at codon 1,291. As we know, the organization of data, including clinical and molecular information, is fundamental to the management of individuals/families at high risk for hereditary cancer.

Starting from 1997, weekly meetings were organized with participation of several professionals interested in hereditary cancer. Then, in February 2003, the Brazilian Study Group on Hereditary Tumors - GBETH was founded. In 2005 and 2007 the group published two updated books on hereditary cancer in Brazil. All the content of these two books is currently available as newsletters on www.geth.org.br. In 2007, with these publications and with the participation of the group in international meetings, professionals from other countries increased, mainly those from Argentina, Chile and Uruguay, became interested to participate. So GBETH became the Study Group on Hereditary Tumors - GETH (www.geth.org.br), widening its outreach to South America. In 2006, the GETH organized the First International Symposium, with the presence of several international guests and more than 170 registered participants.

Currently, the GETH website is one of the major tools for the integration of group members. The website has a restricted area for members, accessed only with login and password, which allows entering a forum with clinical cases, thematic panels on hereditary cancer, as well as giving access to all group meetings held from 2014, which have been recorded in video, as well as the newsletters written and published during the last years. In the future, we plan to perform the meetings with recordings and translation from Portuguese to Spanish, through captions, which breaks the language barrier in the dissemination of information.

The GETH performs periodic meetings at the Sirio-Libanês Hospital in São Paulo, which boasts a very well organized and structured convention center that allows for live broadcast on web streaming and simultaneous recording in high definition, so that subsequent access by those interested is possible. Live access can be made by streaming, via the WEB, through any computer or even mobile phone, in real time, after registration on the teleconference system of Sirio-Libanês Hospital. The possibility of distant participation is fundamental in Brazil and South America, as the institutions are located far apart, with different technological structures and variable financial support.

The project which the GETH is currently working on is to establish the South American Collaboration of Registries on Hereditary Cancer on a WEB platform. The idea is to use non-proprietary software on a WEB platform to register the data of families with suspected hereditary predisposition to cancer. This tool is being built by the Engineering/Computer Science Department from the University of São Paulo (already in functional testing and security) with the goal of each project participant having individualized access to his/her own data via login and password. Thus, the information from each institution is safeguarded against access by other participants in the collaboration. Even hierarchical access within the same institution can be achieved, according to pre-determined decisions by the participants of the respective project. Nonetheless, it is essential that all collaborators of the Registry on the WEB platform have the same clinical and molecular data for possible future research or clinical collaborations.

Another benefits that the system offers is distant access via internet from any computer or mobile phone, using a standardized data storage system for participants of the South American Collaboration of Registries on Hereditary Cancer.

On September 21, 2014, the South American Workshop of Hereditary Cancer - WSACH 2014, was held in the Sirio-Libanês Hospital in São Paulo, Brazil. The main objective of this meeting was to bring together the leaders from Brazil and South America in the area of Hereditary Cancer to a large panel in order to start the South American Collaboration of Registries on Hereditary Cancer. It was an invitation-only event, with 70 participants representing 35 different institutions/universities from all South America.

This whole process of network construction and research development on Hereditary Cancer in Brazil and in South America prepares the base for global collaborations, mainly with the International Society for Gastrointestinal Hereditary Tumours – InSiGHT (www.insight-group.org) and the Collaborative Group of the Americas – Inherited Colorectal Cancer – CGA-ICC (www.cgaicc.com). The most important objective of this South American initiative is to consolidate the area of hereditary cancer registry and research all over the continent.

